# The cultural ecology of social media

**DOI:** 10.1017/ehs.2026.10052

**Published:** 2026-05-29

**Authors:** Alberto Acerbi

**Affiliations:** Department of Sociology and Social Research, University of Trento, Trento, Italy

**Keywords:** cultural evolution, cultural ecology, cultural transmission, misinformation, social media

## Abstract

Most research on social media considers them as supports for information transmission, explaining online success (and pathologies) by focusing on consumers’ biases and interests. This article takes a different perspective, applying ideas from an ecological approach to culture. Success online depends both on the intrinsic appeal of content to receivers and on how well content serves producers’ strategic goals within the constraints and affordances of specific platforms. These goals include reputation management, coalition building and identity management, and coordination or participation in shared activities. Transmission is often a by-product of these motivations, and replication fidelity plays a limited role compared with transformations that adapt content to local incentives. Finally, the article suggests that platforms and communities can be understood as distinct niches, each characterised by different audience structures, affordances, metrics, and algorithmic pressures. This perspective reframes persistent debates on social media dynamics, including misinformation, radicalisation and polarisation, and the reasons behind online success.

## Graphical abstract

**Figure:**
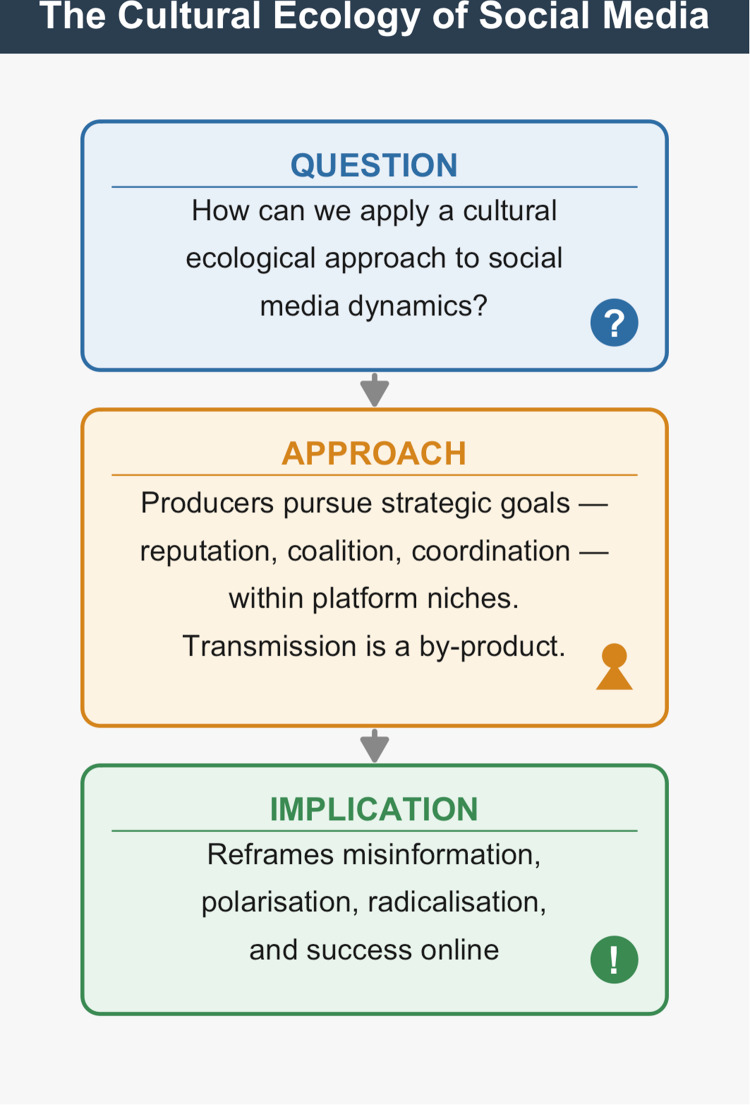

## Social media summary

Cultural dynamics on social media as adaptive strategies: incentives and niches beyond transmission models.

## 1. Introduction

Social media research has rapidly expanded across disciplines, from psychology and communication studies to computational social science. Within this field, some approaches have taken a cultural evolution perspective. According to the standard account, cultural evolution theory conceptualises culture as a system of inheritance with similarities to genetics (Boyd & Richerson, 1985; Mesoudi, 2011). Individuals acquire ideas, practices, or preferences through social learning, which is considered faithful enough to implement a process of inheritance. The frequencies of these cultural variants change over time, at the population level, as a consequence of biased transmission and differential adoption at the individual level. Studies in this tradition have examined the effects of context biases, i.e., individual tendencies to prefer certain cultural traits because of contextual factors (such as copying popular traits, or traits that are diffused by prestigious individuals), and content biases, i.e., preferences for intrinsic features of cultural traits (such as particular types of content that make them more attention-catching or memorable).

This framework has been explicitly used by some to understand online dynamics as forms of cultural transmission and selection (Acerbi, 2016, 2019a, 2019b; Acerbi et al., 2023; Barkow et al., 2012; Nickl et al., 2025; de Oliveira & Albuquerque, 2021), while others share similar intuitions without directly adopting the cultural evolutionary background. For instance, various studies analyse online diffusion focusing on which content is appealing to people, such as negative or emotional content (see, for example, references in Rathje & Van Bavel, 2025), in a way similar to content biases in cultural evolution. The central idea is that we should focus on how transmission of information happens on social media and analyse the conditions under which people accept, share, or ignore it (see, e.g., Brady et al., 2023).

Recently, Baumard and André (2025) have argued for studying cultural dynamics through the conceptual tools of behavioural ecology. Rather than seeing culture as a distinct inheritance system requiring an extension of evolutionary theory, they suggest treating cultural phenomena as adaptive responses to ecological conditions, be they physical, social, or informational. This ecological approach emphasises how individuals produce and consume cultural behaviours as strategies to maximise their own specific payoffs (reputation, coordination, coalition building) given local constraints and opportunities.

This article applies this perspective to the study of social media. While the broad implications of an ecological approach to culture, and its relationships with the more traditional cultural evolution theory, are outside of my scope, I suggest that thinking of social media in ecological terms allows us to examine their informational dynamics from a more productive angle. The question is not primarily how information spreads, but why people produce and share it in the first place, and what functions these behaviours serve in their respective online environments.

The question of what people do with media, and to what ends, has been raised before. Uses and gratifications research (Katz et al., 1973) made a structurally similar move and catalogued a range of motivations: information seeking, identity expression, entertainment, social interaction. The ecological approach proposed here shares this reorientation but differs in two respects. Rather than inductively listing user needs, it grounds motivations in evolutionary reasoning about fitness payoffs, asking which goals should be prevalent and why. It also connects producer motivations more directly to the specific constraints of platforms: platform studies has extensively described how affordances and algorithmic architectures vary and shape user behaviour (Bucher & Helmond, 2018; Gillespie, 2010), but has not typically connected these structures to the incentive logic that makes particular content traits adaptive in particular niches. The ecological framing attempts to bridge these two levels of analysis.

In what follows, I outline the elements of a cultural ecology of social media. First, I discuss the role of content producers, emphasising that to understand why some content is successful on social media we need to look at the features that make it so for producers’ goals, such as reputation management or coalition building. Second, I argue that transmission is often a by-product rather than the primary aim of online behaviour, and that replication, in the Darwinian sense, plays a limited explanatory role. Even if technically possible and easy to implement, online content is mostly transformed, and it persists or vanishes because of how it fits individual incentives and affordances of platforms. Finally, I suggest interpreting different social media platforms, as well as communities within those, as distinct ecological systems, each characterised by its own constraints, resources, and selective pressures. This framework, I argue, can also shed a different light (and suggest different empirical approaches) on persistent social media issues, such as misinformation, polarisation, algorithmic radicalisation, or the reasons for online success.

## 2. Towards a cultural ecology of social media

### 2.1. The role of producers

Every day, on social media, millions of people produce and share information, in the form of written posts, images, videos, or reviews of any imaginable product or experience, often with no measurable return. From a cultural evolution perspective, this behaviour may appear paradoxical (Acerbi, 2019b): information producers pay a personal fitness cost that allows social learners – information scroungers – to benefit from their exploration (Rogers, 1988).

An ecological approach to culture reverses the issue by asking what goals individuals pursue when they produce and circulate cultural items (Baumard & André, 2025). The distinction matters, because it shifts the explanation from the properties that favour the spread of a cultural trait to the incentives that shape its production. Traditionally, cultural evolution has explained the properties of cultural traits by considering characteristics of the receivers or consumers of the traits. The ‘producers’ standpoint’ (André et al., 2023) suggests instead that the properties of cultural traits reflect (also) the payoffs that individuals expect to obtain. To illustrate, from a consumer-focused perspective, one might conclude that content about possible threats spreads widely because more likely to capture attention and be retained. A producer-based interpretation suggests instead that individuals often create or share material about possible threats because it is effective for their goals, such as signalling alertness or competence (Blaine & Boyer, 2018; Boyer & Parren, 2015).

Attracting attention can be considered a meta-goal for any other activity. Attention is quantifiable on most platforms. Producers can see in real time how many people have viewed, liked, or shared their posts, and these metrics constitute a salient reward structure. Lindström et al. (2021) showed that we are sensitive to these rewards, and we calibrate our posting activity to maximise engagement signals (for a similar approach, see Turner et al., 2025). Attention-oriented content typically displays universal features that, on average, facilitate engagement from the consumer point of view: high-arousal emotion, surprise, humour, provocation, or novelty, which have been often studied. The often-noted success of negative content fits this logic (Baumeister et al., 2001). Brief formats, such as short videos, punchy statements, and concise images, are effective because easier and faster to process, and producers who quickly capture attention have the additional advantage of being likely rewarded by platform algorithms.

However, more specific predictions can be made. First, different platforms, and different communities within platforms, can reward different content (see ‘Different platforms as different ecologies’ section). Second, different content is linked to distinct goals and incentives. We will analyse some of them: reputation management, coalition building and identity management, participation in shared activities and coordination. These goals do not represent an exhaustive list, but they capture the main types of incentives that drive production in social media. Importantly, they also allow us to infer the features we should expect successful content to have.

From this perspective, cultural patterns on social media are not only, or primarily, the result of biases that govern how consumers acquire information. They are shaped by the strategies that producers adopt, and what becomes widespread is what best serves these strategies. Here, ‘production’ covers every action that can be performed on social media, with a different level of engagement: from creating and posting novel content, including comments, to sharing content produced by others, or endorsing it (with a ‘like’ or similar). In this sense, the distinction between producers and receivers is used as an analytical convenience rather than as a claim about distinct categories of individuals: all users are strategic agents. In fact, the case of social media is particularly interesting exactly because productive acts – like posting, sharing, or liking – are not only cheap and available to virtually all users but also persistent and potentially visible to large and heterogeneous audiences.

#### Reputation management

The first set of goals concerns reputation management. Individuals benefit from being regarded as competent, trustworthy, vigilant, or committed (but also as funny, witty, or likeable), and many forms of cultural production can be understood as attempts to manage how one is perceived by others (Mercier, 2022). Social media amplify these processes by making social performance persistent and easily comparable. Posts, images, and comments become tools for the strategic presentation of self: individuals highlight desirable traits and suppress unfavourable ones, tailoring their contributions to the audiences they expect to encounter.

Sharing activity, for example, does not necessarily imply that we are interested or engaged with a social media post, but that we want to signal that we are interested or engaged. Comparing news reading and sharing on the BBC website, Bright (2016) found no correlation (in fact, a small negative correlation) between categories of read and shared articles. ‘Social Welfare’ and ‘Science and Technology’ categories were the most shared, signalling competence and commitment, but the most read category was ‘Accident and Disaster’. Individuals may share political news to justify their own beliefs (Williams, 2023) or to signal commitment to group values (see below) without subscribing to their specific content. Within this perspective, informational accuracy is only one of the possible strategies to manage reputation. Novel or distinctive contributions can signal creativity, and taking provocative or risky positions can be advantageous when the potential reputational payoff exceeds the social cost.

#### Coalition building and identity management

Reputation does not exist in the void. People do not signal competence, morality, or vigilance for an abstract audience; they signal to specific groups whose approval, trust, or support they seek. This connects reputation management with a second set of goals, centred on coalition building and identity management. Humans are highly sensitive to group boundaries, and many forms of communication function to mark allegiances, indicate shared values, or maintain membership within a community (Tooby & Cosmides, 2010). From an ecological standpoint, producing content that signals coalition membership can be advantageous because it secures support, reduces uncertainty about one’s intentions, and clarifies one’s social position.

Content that serves these goals tends to display characteristic features. Negative evaluations of out-groups or strong affirmations of shared norms are predictable when individuals benefit from clarifying their allegiances. Moral and political statements often act as group markers rather than attempts at persuasion. The prevalence of highly moralised or polarised content online, which is generally interpreted as evidence of cognitive or emotional biases on the receiver side (Brady et al., 2017), can also be seen as a rational outcome of producers using content to secure coalitional advantages. In this perspective, moralised content should be more likely to be produced and circulated in contexts where social identity is salient and where group boundaries are contested.

Social identity management operates alongside coalition building. People use social media to express who they are and to signal alignment with particular cultural styles, lifestyles, or communities. Identity-oriented content must be easily recognisable and difficult to misinterpret. It relies heavily on symbolic markers: aesthetic conventions, references to shared experiences, specific forms of humour or irony, and visual cues that situate the producer within a recognisable group. The proliferation of meme genres or group-specific vocabulary can be explained by producers’ motivations to manage their social identity, possibly stronger when online identity is an important form of their social status, as in marginalised online groups (Costello & Thomas, 2025).

An interesting special case concerns producers whose primary incentives are monetary, e.g., X ‘creators’. In these cases, reputation and identity management are directed at the creation of a persona built to maximise visibility and retention. Content creators who depend on advertising revenue, subscriptions, or sponsorships have strong incentives to optimise directly for audience engagement, but their strategies differ depending on how monetisation is achieved. In general, monetised producers should be more responsive to engagement metrics and adapt more quickly to platform trends. Producers relying on broad reach are likely to display weaker sensitivity to local norms or niche-specific constraints, while those monetising through subscriptions or patronage may, on the contrary, be highly sensitive to the norms and expectations of a specific audience. Although monetised producers likely represent a numerical minority, their optimisation strategies may grant them disproportionate visibility. This distinction generates testable predictions, for example, by comparing monetised and non-monetised accounts, or different monetisation models, in terms of content diversity, responsiveness to metric feedback, and convergence or divergence across niches.

#### Participation in shared activities and coordination

A third set of goals concerns participation in shared activities and, in some cases, the coordination of collective behaviour. Humans are strongly motivated to engage in joint action and to maintain social presence within their groups, even in low-stakes settings (Tomasello et al., 2005). Much everyday social media production consists of joining ongoing conversations and trends, not because the content itself is informative, but because participation signals affiliation and reduces the cost of social interaction. Typical examples include the replication of meme templates, the adoption of a trending audio or video on TikTok, or participation in chain prompts on Instagram. These formats succeed because they provide a recognisable structure into which users can insert minimal personal variation. From an ecological perspective, such content spreads not due to intrinsic features but because it enables individuals to take part in collective practices at low cognitive and reputational cost (Berriche & Altay, 2020).

Coordination-oriented behaviour forms a smaller proportion of total production but illustrates the same logic. People use social media to organise demonstrations, coordinate donations, report local incidents, or mobilise support for campaigns (Heverin & Zach, 2012; Vieweg, 2012). Effective coordination signals must be clear, unambiguous, and easy to share; they reduce uncertainty and facilitate group-level action. Empirical work on protest mobilisation and natural disasters shows that social media activity often spikes when timely, actionable information is needed, and that these messages exhibit characteristic features, such as directives, timestamps, and concrete instructions.

### 2.2. Transmission and replication

Drawing on what I discussed above, the primary aim of social media activity is rarely transmission. While this could be the case for specific goals, like participation in shared activities or coordination, transmission is often a by-product of other motivations rather than a goal in itself. For this reason, when we study informational dynamics on social media, we may want to shift our analytical attention from the mechanics of transmission to the goals that generated it.

A case that is probably familiar to readers is the use of social media by academics. When academics post information about their research, links to their papers and preprints, or conference talks, they do produce and transmit information, but they are not uninterested information producers. To understand this dynamic, we cannot focus only on receivers’ biases, but we need to know the motivations behind sharing, generally self-promotion. Even when academics share works from other researchers, producing useful information, their goal may be to build their reputation as competent scholars in a field and to let colleagues know that they are up-to-date on a particular topic. In most cases, they will share information that is consistent with their perspective, but they could gain extra reputation by sharing and commenting on different views. Generally, there will be a strong incentive to share true information, as the academic community will be able to assess its quality, and reputation damage could be costly.

It can be useful, as an exercise, to consider an illustrative case where visibility differences might appear to reflect receiver bias. For example, women’s papers tend to be less present or less engaged with on social media. A transmission-based interpretation would attribute this to audiences being less inclined to attend to or share work authored by women. Evidence from Peng et al. (2025) points to a producer-level mechanism instead: women are substantially less likely than men to promote their own papers online, even after controlling for academic status and productivity. In this example, differences in diffusion patterns emerge not because receivers prefer certain authors, but because producers differ in how they use social media.

Relatedly, even if digital transmission offers a cheap and virtually error-free possibility of replication that would nicely fit with Darwinian-inspired models of transmission, social media dynamics make explicit that replication fidelity has little explanatory power by itself. Transmissibility is effectively constant across cultural items, so the relevant question becomes why individuals choose to replicate one item rather than countless others that are equally easy to copy and reproduce.

Moreover, cultural dynamics on social media often result from transformation, reframing, and embedding in new contexts. This can be clearly seen by analysing the diffusion of internet ‘memes’, whose name suggests Darwinian-like replication (for a longer discussion of non-replication of memes, see Acerbi (2019b, Chapter 7)). In an early study on ‘information evolution’ on social media, Adamic et al. (2016) considered Facebook memes from 2009 to 2011. At the time, Facebook did not have a built-in ‘share’ button, so text needed to be copied-and-pasted manually. Successful memes, replicated verbatim, existed: ‘No one should die because they cannot afford health care and no one should go broke because they get sick. If you agree, please post this as your status for the rest of the day’ was found in the exact same form more than 470,000 times. However, this was rather the exception: the ‘mutation rate’ of transmission was, surprisingly given the digital support, extremely high. On average, 11 out of 100 copies were different from the original.

These differences, moreover, were not random. Some were clearly deliberate innovations. Common variants of the text above included ironic takes (‘No one should be without a beer because they can not afford one’) or appeals to the opposite political spectrum (‘No one should go broke because government taxes and spends …’). Others were likely due to psychological or technical constraints, with shorter (but not too short) sentences being copied more faithfully, or differences and omissions being more likely to be found at the beginning or at the end of texts. Because of the prevalence of these transformations, the researchers considered as a ‘meme’ not a discrete cultural token but a family of variants held together by partial resemblance, similarly to the concept of cultural attractor (Miton, 2025).

In sum, the spread of internet memes can hardly be modelled as a process of reproduction, random mutation, and receivers’ biases. Memes became associated with coalition building and identity management goals independently of their intrinsic features (e.g., ‘Pepe the Frog’). Others are templates for mimicry or remix (Shifman, 2013). Historical memes like ‘Grumpy Cat’ or ‘Jealous Girlfriend/Distracted Boyfriend’ owe their success to the possibility of endlessly editing their captions.

This does not imply that transmission is irrelevant in social media dynamics. What spreads must still be intelligible and appealing enough to be picked up by others, and many producer-driven contributions would fail without some degree of transmissibility. The point is rather that transmission does not operate in isolation. High-fidelity replication is only one of several pathways through which information can circulate, and often not the dominant one.

### 2.3. Different platforms as different ecologies

Rather than treating ‘social media’ as a homogeneous domain, an ecological view emphasises that each platform creates a different landscape of incentives for cultural behaviours. Platforms differ in audience structure, affordances, feedback metrics, and algorithmic distribution mechanisms, and these differences systematically shape the kinds of cultural items that are produced and how they change over time.

#### Audience structure

Audience structure is one of the fundamental parameters shaping cultural production online. Online communication, and social media in particular, has increased enormously the number of individuals with whom we can exchange information, while at the same time making this audience opaque (Acerbi, 2019b). However, producers rarely address an undifferentiated public; instead, they act according to their imagined audience, a mental model of who is watching and how they might react (Litt, 2012). Different platforms elicit different models. On platforms such as Facebook, for example, where audiences are often anchored in offline social ties, users adapt their self-presentation to a mixed public of friends, colleagues, and family members, generally favouring more generic and cautious self-disclosures, stronger attention to perceived norms of appropriateness, and a lower propensity to post contentious political content, as studies of disclosure patterns and self-censorship suggest (Burnett et al., 2022; Gil-Lopez et al., 2018).

In contrast, say on X, where audiences are largely unknown, mostly composed of weak ties, and posts can potentially spread far from the circle of contacts, producers may orient their content to imagined communities of peers or ideological allies. These patterns operate within, and may reinforce, clustered audiences that have been found in analyses of social media like Twitter (Barberá, 2015; Conover et al., 2011). Homophilous audiences reward clear identity boundary markers, partisan signalling, and moralised framings, even when these reduce cross-group reach. Conversely, one may expect that in specialised or professional communities, such as the social network LinkedIn, with relatively cohesive audiences, informational accuracy, demonstrations of expertise, and community-specific norms will be rewarded (Zhong et al., 2017).

#### Affordances

Affordances define what users can do on a platform and how easily they can do it. Features such as quote-tweets, duets, stickers, filters, reaction emojis, hashtags, or character limits make some actions intuitive and others cumbersome, guiding producers towards specific communicative strategies. For example, TikTok’s tools for audio reuse and video remixing encourage iterative mimicry, generating lineages of content that evolve through small modifications rather than replication (see ‘Transmission and replication’ section). Analysing almost two million political videos, Guinaudeau et al. (2022) show that TikTok’s short-form format and low production barriers generate a different communicative environment with respect to YouTube, with a higher proportion of TikTok users uploading videos, suggesting that creation tools and remix-friendly features lower participation costs.

Ephemeral formats, such as Snapchat messages or Instagram Stories, reduce the long-term reputational risks of communication, making disclosures less costly. Research on Snapchat use shows that disappearing content encourages more spontaneous, informal, and self-expressive communication, precisely because it is less likely to be taken out of context or have repercussions across different social audiences (Bayer et al., 2020).

A contrasting affordance is provided by platforms that feature long-form written content, such as Substack and other newsletter ecosystems. Here, the possibility of producing extended, persistent posts creates a selective environment different from short-form, remix-driven platforms. While there are no studies on modern newsletter ecosystems, it is likely that rather than rewarding rapid adaptation to trends, long-form formats privilege sustained argumentation, thematic continuity, and demonstrations of expertise. Because audiences subscribe and return over time, producers have incentives to invest in accuracy and narrative coherence, and the persistence of posts reduces the appeal of ephemeral or trend-driven content.

#### Feedback metrics and algorithmic distribution mechanisms

Feedback metrics such as likes, shares, comments, and view counts quantify audience reactions and provide producers with rapid signals about which traits are rewarded. Algorithmic distribution systems amplify these incentives by determining which content becomes visible beyond one’s immediate network. On TikTok, early engagement strongly predicts wider diffusion, and visibility depends only weakly on account size. The same study comparing YouTube and TikTok political videos (Guinaudeau et al., 2022) shows that TikTok’s recommendation system enables small accounts to achieve substantial reach when their content aligns with algorithmic preferences. Algorithms can also alter recency rankings: in 2025, LinkedIn introduced a feed update that resurfaced posts several weeks old, aiming to prioritise perceived relevance over chronology. Such mechanisms extend the lifespan of content and may incentivise producers to create material that remains valuable beyond the moment of posting.

Platforms differ in whether their ranking systems privilege social ties or global virality. Facebook and LinkedIn rely mostly on network-based distribution, where posts from friends, colleagues, or subscribed pages dominate the feed; producers in these environments benefit from tailoring content to the expectations of stable, known audiences. By contrast, TikTok and Instagram Reels rely largely on interest-based ranking, where visibility depends on engagement potential rather than interpersonal connections. These contrasting logics create distinct selective pressures: network-centric systems reward content calibrated to local norms, while virality-centric systems favour broad appeal and formats suited to algorithmic discovery.

#### Niches within platforms

Ecological differences do not arise only between platforms but also within them. Large social media systems host multiple communities that operate as distinct niches, with different norms and expectations, and thus selective pressures on cultural traits. On X, for example, political clusters reward partisan signalling and moralised framings (Brady et al., 2017). However, other communities on the same platform may follow different incentives. In a recent study of archaeologists’ communication on X (Bonacchi et al., 2025), positive updates and professional achievements were more successful than negative or confrontational content, suggesting that cohesive, occupationally defined groups favour expertise and collegiality rather than outrage. The same affordances and algorithms thus support distinct cultural dynamics depending on the goals and expectations of the local audience. More generally, evidence suggests that positivity can thrive in the right contexts, for example, when the large majority of content is negative, or, as in the case mentioned here, in specific niches (Soroka & Krupnikov, 2021).

A similar contrast emerges when comparing social media dynamics with settings where audiences are smaller and more relational. Research on New York Times email sharing showed (again in contrast with the general success of negative content on social media) that positive, high-arousal stories are more likely to be forwarded to close contacts, reflecting the social goals of relationship maintenance and supportive signalling (Berger & Milkman, 2012). Within large, anonymous, or ideologically structured niches, producers face different incentives: signalling vigilance, commitment, or group identity becomes more adaptive than sharing uplifting material. These examples illustrate that platforms are not single ecologies but constellations of smaller ones, each characterised by distinct selective pressures. Understanding social media dynamics could therefore require analysing cultural dynamics at the level of these niches rather than treating platforms as homogeneous environments.

## 3. Thinking differently about social media issues

### 3.1. Misinformation

One of the biggest concerns that accompanied the diffusion of social media has been that they would provide a fertile ground for the spread of misinformation. More recently, many assumptions behind this idea have been challenged. For example, while some features of social media transmission dynamics could favour the spread of misinformation, misinformation is not a social media-specific problem (misinformation from traditional media and, especially, from public figures can be much more impactful). In addition, when compared with the total information present online, or on social media, flagged misinformation represents a tiny minority (Altay et al., 2023).

From the perspective defended here, however, the most interesting point is not the relative quantity of misinformation but the causal model that underlies most research on the topic. The standard narrative assumes a direct link from exposure to a piece of misinformation to a change in beliefs or behaviour. This causal link cannot be taken for granted. Research shows that ideas and beliefs, especially political ones, are entangled with social and economic factors, and that people rarely change their minds in response to propaganda messages; attitude change is more likely when individuals encounter convincing arguments within trusted relationships (Mercier, 2020). Moreover, if we take the producers’ point of view, misinformation does not spread because people are prone to believe false content, but because producing and circulating certain kinds of information can be useful for particular goals within specific online environments.

From the producer’s standpoint, many forms of misinformation are by-products of incentives that reward attention, identity signalling, or coalition maintenance rather than accuracy. Political misinformation, for instance, is shared almost exclusively by a small subset of individuals who already endorse the views expressed, who also share partisan news from reliable outlets, and who strongly dislike their political opponents (Osmundsen et al., 2021). Emotional, sensational, or norm-violating content is more likely to generate engagement and therefore more likely to be rewarded with visible feedback, whether the content is true or false.

As a consequence, misinformation mostly shares the same features of reliable information. For example, mainstream news online also gathers engagement through divisive and, up to a point, negative content (Fischer & Acerbi, 2025). In theory, a possible difference is that false content can be tailored to maximise attention, while true content is, by definition, more constrained by reality (Acerbi, 2019a). Some research found indeed that, analysing US television broadcasters, inaccuracy and the degree of negativity bias are correlated (Soroka & Wlezien, 2026). However, content-related differences between news produced by reliable and unreliable outlets are likely to be too small to be used to distinguish them, exactly because producers have comparable incentives.

This perspective also changes how we think about interventions against misinformation. Almost all interventions tested so far adopt the consumer’s point of view. Debunking, pre-bunking, nudges, and digital literacy aim to make receivers more sceptical of unreliable sources or false claims. It has already been noted that these interventions have the by-product of making us sceptical of all news, which, given that reliable ones are the majority, can have a marginal negative effect (Altay, 2022). However, what about the producer’s standpoint? Individuals recognise that sharing misinformation can be harmful for their reputation (Altay et al., 2022), and sharing reliable and useful information elicits epistemic gratitude (Karabegovic et al., 2024), a positive social signal that can strengthen trust and make future information exchange more likely.

Interventions, or even platform architectures, could therefore build on these incentives. Rather than focusing solely on reducing exposure or correcting beliefs, platforms could make reputational consequences for accuracy more salient, increase the visibility of epistemically valuable contributions, or design features that reward reliability and domain expertise. The spread of misinformation already varies across platforms and niches: for example, LinkedIn, where professional reputation is at stake, shows lower rates of political content and likely misinformation than platforms organised around anonymous or ideological audiences. This suggests the possibility of shaping environments so that the incentives for producers align less with attention at any cost and more with demonstrating reliability, competence, or usefulness.

Of course, this raises the question of who would actually implement such changes. Platform providers operate under their own incentive structures, primarily optimising for engagement and retention; there is no obvious reason why they would voluntarily redesign architectures to reduce the spread of content that performs well by their own metrics. Meaningful change at the platform level is therefore likely to require external pressure (e.g., regulatory mandates such as the EU’s Digital Services Act) or explicit costs, for example, when misinformation episodes become commercially damaging. A further complication concerns users’ reactions to these changes. If platforms become less rewarding in engagement terms, say, more accurate but less stimulating, users may simply migrate to alternatives that better serve their goals. This competitive dynamic may be as difficult to overcome as the platforms’ own engagement incentives, and is itself a prediction the ecological framework would generate: individuals, like platforms, move towards the niches that best serve their interests.

### 3.2. Radicalisation and polarisation

Concerns about online radicalisation often rely on the same causal model of mainstream research on misinformation: individuals are exposed to extreme content and consequently shift towards more extreme attitudes. Empirical evidence provides a more nuanced picture. Large-scale audits of YouTube’s recommendation system show that while the algorithm tends to supply ideologically congenial material, it does not systematically push users towards more extreme content. In a study using 100,000 sock-puppet accounts, Haroon et al. (2023) find that recommendations become more aligned with inferred ideology, and that exposure to problematic channels remains very limited in absolute terms. Complementary experiments using counterfactual bots demonstrate that relying solely on YouTube’s recommendations actually leads to less partisan consumption than following real user histories, and that sidebar recommendations ‘forget’ earlier extreme viewing patterns (Hosseinmardi et al., 2024). These results suggest that observed radicalisation dynamics stem primarily from users’ pre-existing preferences rather than algorithmic steering.

From an ecological, producer-centred, perspective, polarising and extreme content emerges not from passive exposure but from the incentives operating within specific niches. On YouTube, political creators compete for attention within ideologically coherent communities, and platform affordances may reward provocative and identity-affirming content. Munger and Phillips (2022) describe this as a ‘supply-and-demand’ dynamic, where committed audiences create a market for fringe producers and the affordances of the platform facilitate production at scale. Similar logics apply across social media: in homophilous political clusters, producers benefit from content that marks boundaries, signals loyalty, or displays moralised outrage. Polarisation, therefore, reflects the selective pressures of particular online ecologies and producers’ interests rather than a universal susceptibility to extreme messages.

An ecological perspective also explains why broad claims about algorithmic polarisation often overstate the empirical case. Evidence suggests that patterns of hostility or extremity observed online frequently reflect dispositions that predate social media use: individuals who behave uncivilly in political discussions do so across multiple online contexts (Mamakos & Finkel, 2023); those preoccupied with social status display hostile political behaviour both online and offline (Bor & Petersen, 2022); and increases in polarisation on platforms such as Reddit have been shown to depend largely on the arrival of already polarised users rather than on the radicalisation of existing ones (Waller & Anderson, 2021). Moreover, offline social environments are themselves far from politically diverse: everyday friendship networks and workplace interactions often exhibit ideological homogeneity (Gentzkow & Shapiro, 2011), residential patterns show marked political segregation (Brown & Enos, 2021), and traditional media ecosystems also display strong partisan alignment, often as pronounced as, or even greater than, that observed on social media (Fletcher et al., 2020; Muise et al., 2022). Much evidence, in sum, suggests that polarisation emerges from deeper social and economic factors and is only reflected online. In particular, polarisation appears most strongly in environments where user composition, platform affordances, and reward structures jointly favour antagonistic signalling, typically within already politicised niches, rather than across social media as a whole. Interventions that focus exclusively on limiting exposure overlook this dynamic. More promising approaches would target the incentives that make polarising content adaptive: reducing engagement rewards for moralised outrage, elevating signals of reliability or crosscutting engagement, or strengthening reputational pressures in anonymous or weak-tie environments.

### 3.3. Success

The arguments outlined thus far suggest that the content that becomes successful on social media may satisfy different constraints, so that no single formula can account for ‘virality’. On one side, as receivers are more likely to engage with content that is easy to process, emotional, negative, and group-relevant, this content will tend, on average, to proliferate online. Social media, after all, are very effective in bringing us the content we want to consume (whether this is the content we should, normatively, consume is another question). Moreover, with a potential audience of billions of individuals connected online worldwide, a viable strategy is to appeal to broad, universal, cognitive factors of attraction.

On the other side, content becomes successful not only because it attracts receivers, but because it affords other producers opportunities to pursue their own goals by resharing or modifying it. These dynamics become even clearer once we consider, as we did, that social media are not uniform environments but collections of informational niches. A content trait that fits the incentives of one niche may fail in another.

Variation across individuals interacts with platform diversity. Not all producers compete along the same dimensions, and different strategies can be simultaneously successful because they appeal to different audiences. Quantitative analyses of engagement patterns consistently show that predictive models of content that generates engagement online have small effect sizes (*R*^2^ ∼ 0.1–0.2 in studies that report it directly, e.g., Schöne et al., 2021, 2023; and considerably lower in others, e.g., Bak, 2026; for studies using other metrics finding small/heterogeneous effects, see, e.g., Brady et al., 2017, 2025; Fischer & Acerbi, 2025; but see, e.g., Rathje et al., 2021; Watson et al., 2024, for moderate effects). While these small effects can have a cumulative impact on millions of messages, they also suggest that heterogeneity arises because users optimise distinct factors of attraction. In political niches, highly moralised or confrontational posts may be rewarded; in informational niches, accuracy and clarity generate engagement; in lifestyle or creative niches, humour and narrative skill may be the most effective strategies. The same platform can therefore host creators who thrive through negative or provocative content and others who succeed through expertise, helpfulness, or aesthetic expression. This diversity in adaptive strategies explains why attempts to isolate a single, universal predictor of virality consistently yield weak or partially inconsistent results: success is relative to the goals of the producers and the ecological constraints of the niches in which they operate. A similar logic comes from research on fiction: narrative success depends on heterogeneous combinations of emotional, stylistic, and thematic elements, with no single factor dominating across works or audiences (Dubourg et al., 2024). Cultural success, in other words, emerges from many possible adaptive strategies, each shaped by how well a trait serves the goals of producers while fitting the dispositions and expectations of the audiences who consume it.

## 4. Conclusion

This article has taken some ideas from an ecological approach to culture (Baumard & André, 2025) and specifically applied them to social media, without attempting to provide a general account of cultural ecology. In other cultural domains, the importance of consumers’ interests may dominate producers’ motivations, or focusing on transmission may be the most fruitful way to understand the phenomena at hand. Perhaps counterintuitively, the specific informational dynamics of social media can explain this difference. In contexts where reproduction is costly or imperfect, such as oral tradition, craft apprenticeship, and early print, the properties of cultural items as replicators genuinely constrain what survives; memorability, compressibility, and copying fidelity all exert selective pressure. On social media, these constraints effectively disappear: any item can be reproduced at zero cost with complete accuracy, so the bottleneck is no longer replication mechanics but the choices made by producers and consumers. Rapid feedback and heterogeneous audiences then make producers’ motivations especially central.

Social media also function as a kind of cultural ‘microscope’, offering a window into cultural dynamics that exist across all domains but are normally difficult to observe directly. In most settings, the strategic motivations behind cultural production leave few, if any, visible traces. Cultural dynamics in social media are not fundamentally different, but they are visible: what people share, how audiences respond, and what content succeeds are all recorded and quantifiable.

As mentioned, it is important to highlight that the producers/consumers distinction is used as a convenient analytical strategy. All individuals can be both, and social media are an interesting environment because actions are unusually amplified and observable, making obvious the importance of production. This framing points towards a deeper implication. The distinction between producers and receivers is strictly linked to a transmission-based view of culture. In a fully ecological picture, there are no producers and receivers but only individuals acting according to their own goals and incentives.

Although largely theoretical, the article suggests various avenues for modelling, prediction, and intervention. A producer-oriented cultural production system can be formally modelled and compared to a more consumer-oriented one to generate predictions about expected informational dynamics, including which content should spread under each regime and how such patterns vary across niches. The analysis also highlights intervention points that could be empirically tested: reputational incentives that discourage misinformation, platform architectures that modulate the visibility of polarising signals, or affordances that promote prosocial participation within particular groups. More generally, the framework offers guidance for future work that seeks to explain online cultural dynamics not only by assuming universal cognitive tendencies or direct exposure effects but by examining the adaptive strategies individuals pursue within the constraints and opportunities of specific platforms and audiences.

## Data Availability

Not applicable.

## Research Transparency and Reproducibility

No data or codes are associated to this research.
